# The effects of extracellular pH and of the transcriptional regulator PACI on the transcriptome of *Trichoderma reesei*

**DOI:** 10.1186/s12934-015-0247-z

**Published:** 2015-04-30

**Authors:** Mari Häkkinen, Dhinakaran Sivasiddarthan, Nina Aro, Markku Saloheimo, Tiina M Pakula

**Affiliations:** VTT Technical Research Centre of Finland, P.O. Box 1000, (Tietotie 2, Espoo), FI-02044 VTT Finland

**Keywords:** *Trichoderma reesei*, Ambient pH, *pac1*, Gene regulation, Gene expression, Carbohydrate active enzymes, Cellulase, Hemicellulase, Transcriptional profiling

## Abstract

**Background:**

Extracellular pH is one of the several environmental factors affecting protein production by filamentous fungi. Regulatory mechanisms ensure that extracellular enzymes are produced under pH-conditions in which the enzymes are active. In filamentous fungi, the transcriptional regulation in different ambient pH has been studied especially in *Aspergilli*, whereas the effects of pH in the industrial producer of hydrolytic enzymes, *Trichoderma reesei*, have mainly been studied at the protein level. In this study, the pH-dependent expression of *T. reesei* genes was investigated by genome-wide transcriptional profiling and by analysing the effects of deletion of the gene encoding the transcriptional regulator *pac1*, the orthologue of *Aspergillus nidulans pacC* gene.

**Results:**

Transcriptional analysis revealed the pH-responsive genes of *T. reesei,* and functional classification of the genes identified the activities most affected by changing pH. A large number of genes encoding especially transporters, signalling-related proteins, extracellular enzymes and proteins involved in different metabolism-related functions were found to be pH-responsive. Several cellulase- and hemicellulase-encoding genes were found among the pH-responsive genes. Especially, genes encoding hemicellulases with the similar type of activity were shown to include both genes up-regulated at low pH and genes up-regulated at high pH. However, relatively few of the cellulase- and hemicellulase-encoding genes showed direct PACI-mediated regulation, indicating the importance of other regulatory mechanisms affecting expression in different pH conditions. New information was gained on the effects of pH on the genes involved in ambient pH-signalling and on the known and candidate regulatory genes involved in regulation of cellulase and hemicellulase encoding genes. In addition, co-regulated genomic clusters responding to change of ambient pH were identified.

**Conclusions:**

Ambient pH was shown to be an important determinant of *T. reesei* gene expression. The pH-responsive genes, including those affected by the regulator of ambient pH sensing, were identified, and novel information on the activity of genes encoding carbohydrate active enzymes at different pH was gained.

**Electronic supplementary material:**

The online version of this article (doi:10.1186/s12934-015-0247-z) contains supplementary material, which is available to authorized users.

## Background

Filamentous fungi can adjust to changing ambient pH of their habitat by an intracellular pH homeostatic system and by controlling the synthesis of gene products directly exposed to the surrounding environment (e.g. cell surface proteins, secreted proteins). Ambient pH regulation of filamentous fungi has been most extensively studied in *Aspergillus nidulans* and has been shown to be mediated by the wide-domain zinc finger transcription factor PacC together with a signal transduction cascade composed of the products of six pal genes (*palA*, *palB*, *palC*, *palF*, *palH* and *palI*) (reviewed by [1]).

Ambient pH is signalled through a plasma membrane complex [2-4]. The components of the pal pathway and of an ESCRT complex are subsequently recruited to the plasma membrane-associated structure. The proposed model for pH signalling can be found from [5]. There are three different forms of PacC in *A. nidulans*. In acidic conditions the full-length form predominates. At alkaline pH, activation of the pal pathway leads to two subsequent cleavage steps resulting in two shorter forms of PacC [6]. The product of the PalB-mediated cleavage step, PacC53, is further cleaved by the proteasome to create the active form of PacC (PacC27) [7]. The active PacC binds to the core target sequence 5′-GCCARG-3′ in the promoters of its target genes [8].

PacC of *A. nidulans* acts as a repressor of acid-expressed genes and as an activator of alkaline-expressed genes [9]. In accordance, null mutations in *pacC* or in the *pal* genes [10,11] lead to a phenotype mimicking that displayed in acidic conditions, whereas a constitutively active PacC results in activation of alkaline-expressed genes and repression of acid-expressed genes regardless of pH [9,10]. PacC controls e.g. genes encoding acid and alkaline phosphatases [10], permeases [12] xylanases (*xlnA* and *xlnB*) [13] and an α-L-arabinofuranosidase (*abfB*) [14]. PacC has also been shown to regulate the expression of the *pacC* gene itself, resulting in an abundance of *pacC* mRNA at alkaline pH [9]. In a recent study, a negative feedback loop attenuating the first cleavage step of PacC during a longer exposure to alkaline conditions was proposed to exist [15].

*Trichoderma reesei* is an industrial producer of especially cellulases and hemicellulases for various applications and is also a widely used host for the production of heterologous proteins [16-18]. In order to create better production strains with enhanced enzyme production properties, the physiological and environmental factors and regulatory mechanisms affecting enzyme production by *T. reesei* have been widely studied. Several transcription factors regulating the expression of cellulase and hemicellulase genes have been identified and characterized in detail (for reviews see [18,19]). The availability of a complete genome sequence of *T. reesei* has made it possible to use genome-wide methods to study the various factors affecting protein production [20]. However, the effect of ambient pH on the production of cellulases and hemicellulases has received less attention, although previous studies indicate efficient production of xylanases at high pH and cellulases at low pH [21]. Recently, endoglucanase production by *T. reesei* was suggested to be highest at pH4.5, whereas exoglucanase and β-glucanase production reached their highest values at pH5 and 5.5, respectively [22]. However, another recent study suggests that there are differences in the optimal pH for cellulase and hemicellulase secretion between different *T. reesei* strains [23].

In this study, effects of the changing ambient pH on the transcriptome of *Trichoderma reesei* were studied by transcriptional profiling. The pH-responsive genes were screened from the genome using the transcriptome data and classified in order to identify the major gene groups affected by the extracellular pH. In addition, the effects of the regulator PACI were studied by constructing a deletion strain. The effects of the changing pH conditions on the members of specific gene groups were investigated in more detail. These genes included the ones encoding carbohydrate active enzymes, genes encoding the components of the pH signal transduction pathway and known and candidate regulatory genes of cellulase - and hemicellulase-encoding genes. The transcriptome data also enabled identification of co-regulated genomic clusters containing especially secondary metabolism genes.

## Results

### pH responsive genes of *T. reesei* according to microarray analysis

A recombinant strain from which the *pac1* open reading frame had been replaced by a hygromycin resistance cassette was constructed. This *pac1* deletion strain (designated as ∆*pac1*) was cultivated in a bioreactor on a medium containing Avicel cellulose at pH6 in parallel with the parental strain QM9414. In addition, the parental strain QM9414 was cultivated in a bioreactor on the same medium in different pH conditions (pH3, pH4.5 and pH6). Three biological replicates of the strain QM9414 and ∆*pac1* were cultivated in each case, and samples collected from two different time points of each culture were subjected to transcriptional analysis using the microarray method. The microarray platform used in this study includes probes for the transcripts of approximately 10 000 genes, including genes from the genome versions 2.0 and 1.2 [24,25], and for putative novel genes detected in a previous study [26]. Statistical analysis of the differentially expressed genes at different pH and in comparison of the cultures of the ∆*pac1* strain and its parental strain was carried out using Limma (R, bioconductor [27,28]) with a fold change log2 > 0.4 and a p-value < 0.01 as a threshold.

**Figure 1:**
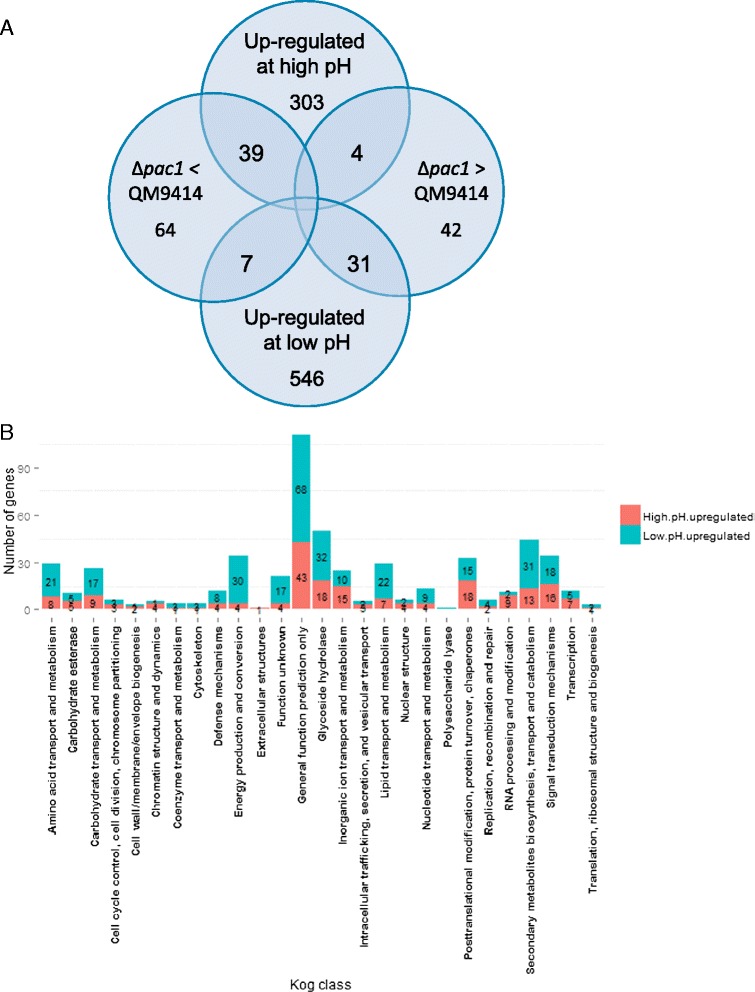
pH-responsive genes of *T. reesei.* **A**, Venn diagram showing the number of differentially expressed genes in comparison of the Δpac1 strain and its parental strain QM9414 at pH 6, as well the number of genes up-regulated in low pH conditions or in high pH conditions. (The number of genes with a significant difference in expression, p value < 0.01 and fold change log2 > 0.4, detected at both time points of the triplicate cultures). **B**, Functional classification of the genes with differential expression in different pH conditions. Classification is based on Eukaryotic orthologous groups. Glycoside hydrolase, carbohydrate esterase and polysaccharide lyase genes were annotated according to [30]. The genes that are up-regulated at high pH are designated as “High pH up-regulated”, and the genes up-regulated at low pH are designated as “Low pH up-regulated”.

The statistical analysis revealed that in the strain QM9414 the expression of 940 genes (~9% of the transcripts in the array) responded significantly to the change of pH in pair-wise comparisons between pH6 and pH3 and/or pH6 and pH4.5 and/or pH4.5 and pH3at both the time points analysed (for details, see Additional file 1). 346 genes were up-regulated at high pH (expression increased significantly in a comparison between a higher pH and a lower pH) and 584 were up-regulated at low pH (expression decreased significantly in a comparison between a higher pH and a lower pH) (Figure 1A). In addition, eight genes had a positive response to the change of pH from 3 to 4.5 but a negative response to the change of pH from 4.5 to 6, and two genes showed the opposite expression trend having the lowest signal at pH 4.5.

In total 189 genes (~2% of the transcripts in the array) were differentially expressed in the cultures of the ∆*pac1* strain as compared to the parallel cultures of the parental strain at pH6 (Additional file 2). Of these genes 77 were up-regulated and 112 down-regulated in the deletion strain as compared to the parental strain. Distribution of genes showing differential expression either at different pH and/or in the ∆*pac1* strain as compared to its parental strain is shown in the Figure 1A. The gene set was explored for the ones most likely to be under direct PACI regulation using the Limma analysis of differential expression and based on the knowledge in PacC-mediated regulation in *Aspergilli*. The PacC transcription factor of *A. nidulans* is activated at alkaline pH. The active regulator represses the transcription of acid-expressed genes and activates the transcription of alkaline-expressed genes [9]. Deletion of the *pacC* gene leads to a phenotype mimicking acidic conditions. Therefore, the hypothesis was that the *T. reesei* genes directly repressed by PACI are only active at acidic pH and thus are more highly expressed in the parental strain at pH3 as compared to pH6. In the *pac1* deletion strain, gene expression mimics that of the parental strain in acidic conditions and therefore the expression of the PACI repressed genes is higher as compared to the parental strain when grown at pH6. Similarly, the *T. reesei* genes induced by PACI are active at alkaline pH and thus are more highly expressed at pH6 as compared to pH3 in the parental strain. In the deletion strain, expression of the PACI-induced genes is lower as compared to the parental strain grown at pH6. According to these criteria, 30 and 38 genes were found to be repressed and induced by PACI, respectively (for details, see Additional file 2). When the expression data was clustered using the Mfuzz clustering method [29], the majority of the genes activated by PACI were assigned to a common Mfuzz-cluster whereas the PACI repressed genes were more evenly divided to several clusters.

The binding site of *T. reesei* PACI in the promoters of its target genes has not yet been characterised. However, the binding sites of the orthologues in *A. nidulans* and in the yeast *Saccharomyces cerevisiae* have been identified, and the core binding sites found to be very similar, GCCARG [8], suggesting conservation of the motif in evolution. The presence of the binding motif GCCARG in the promoters of *T. reesei* was tested. DNA sequences 1500 bp upstream from the gene start site (the start sites as in [24]) were analysed for the presence of the motif. The motif was significantly enriched (p < 0.01) in the set of genes that are likely to be under PACI regulation. 78% of the genes (53 out of 68 genes) harboured the motif. Especially, the motif was enriched in the set genes that are candidates for PACI-induced genes, 87% of the genes containing the motif (33 out of 38 genes, p < 0.0025). However, the motif is frequent in the *T. reesei* genome. 65% of the genes harbour the site in their promoters.

### Classification of the pH-responsive genes

**Figure 2:**
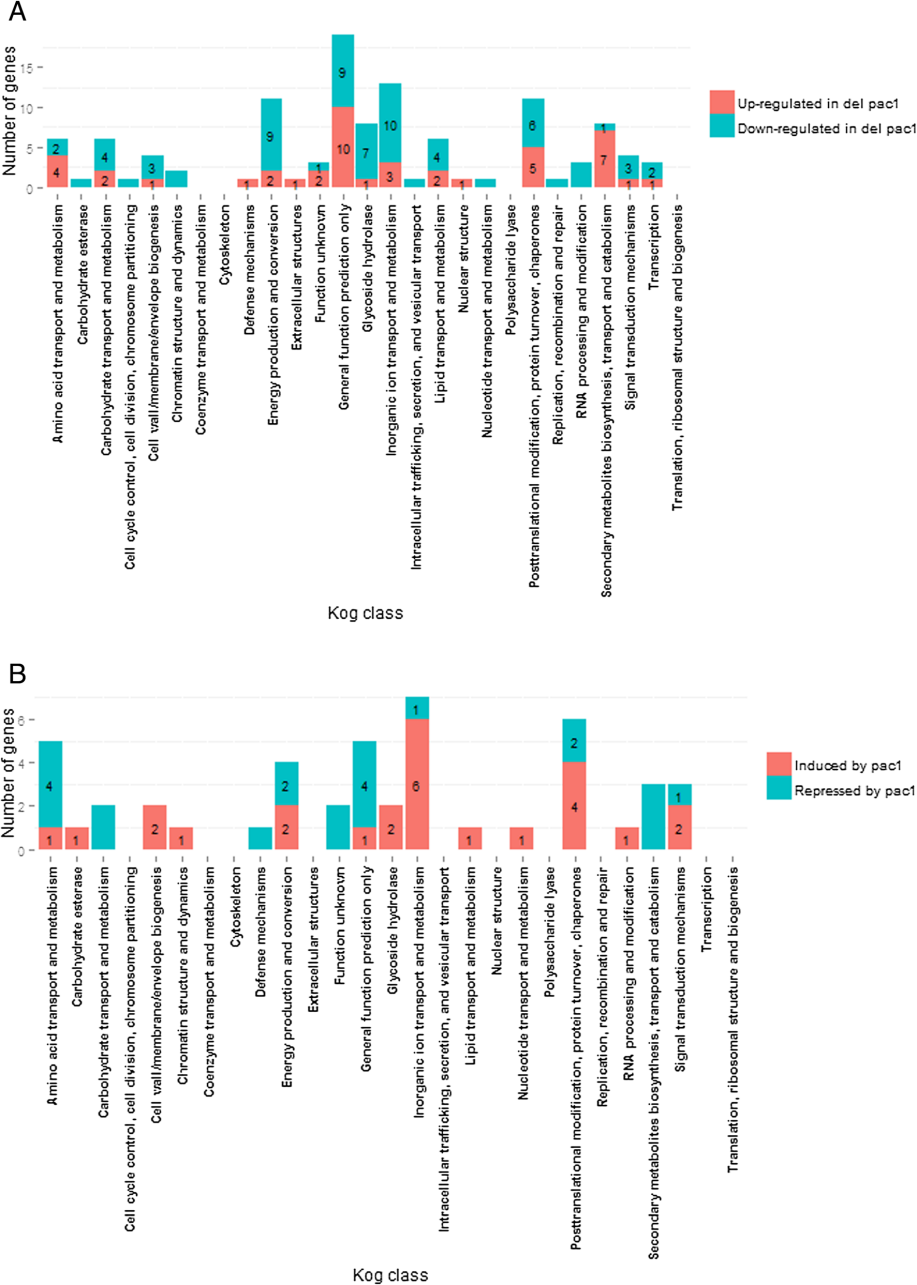
Functional classification of the genes affected by the PACI transcription factor. **A**, genes that are either up-regulated or down-regulated in the *pac1* deletion strain as compared to the parental strain QM9414. Blue bars indicate the number of genes up-regulated in the Δpac1 strain as compared to the parental strain, and red bars the number of genes down-regulated in the Δpac1 strain as compared to the parental strain; **B**, candidate genes under PACI regulation according to statistically significant difference in expression at different pH and in comparison of the parental strain and the ∆pac1 strain Classification is based on Eukaryotic orthologous groups. Glycoside hydrolase, carbohydrate esterase and polysaccharide lyase genes were annotated according to [30]. The genes that are up-regulated at high pH are designated as “High pH up-regulated”, and the genes up-regulated at low pH are designated as “Low pH up-regulated”.

The pH-responsive genes were divided into different functional classes according to the Eukaryotic orthologous groups (KOG) classification (Figure 1B). The genes encoding glycoside hydrolases, carbohydrate esterases and polysaccharide lyases were classified according to the updated annotations [30]. A substantial proportion of the pH-responsive genes was classified as having a general function prediction only, an unknown function or did not have a KOG or CAZy (carbohydrate-active enzymes, http://www.cazy.org/) classification. A putative function could be assigned to a number of these genes according to the manual annotations in the Joint Genome Institute (JGI) *T. reesei* v2.0 databank [24] or according to functional InterPro domains. Classification of the pH responsive genes is presented in Additional file 1. Analysis of the differentially expressed genes in the Δ*pac1* strain as compared to the parental strains revealed genes essentially from the same functional groups as the pH-responsive genes (Figure 2A). However, the proportion of candidate PACI-regulated genes varies within the functional classes. The functional groups “Inorganic ion transport and metabolism”, “Posttranslational modification, protein turnover, chaperones” and “Amino acid transport and metabolism” contain the highest percentage of candidate PACI-regulated genes (28%, 18%, and 17% of the pH-responsive genes in the class, respectively), whereas the glycoside hydrolase class, which is the most abundant pH-responsive functional class, contains only 4% of candidate PACI-regulated genes. Classification of the genes affected by the *pac1* deletion and the candidate target genes for PACI-mediated regulation is presented in Figure 2A and Figure 2B, respectively (for detailed information, see Additional file 2).

### The effect of pH on the components of the pH-signalling pathway

**Figure 3:**
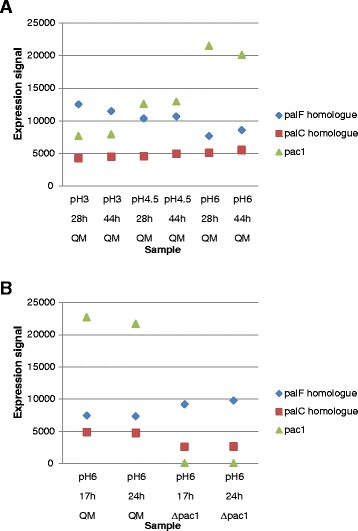
Expression profiles of the gene *pac1* and the homologues of *palC* and *palF* genes in the microarray data. **A)** Expression of the genes in cultures of the strain QM9414 at pH 3, 4.5 and 6. **B)** Expression of the genes in cultures of the Δ*pac1* strain and in QM9414 at pH 6. Expression level of the genes is the normalised signal in the microarray data (not log2 transformed values), data on the three biological replicates is averaged. The time point and pH of the culture, as well as the strain, is indicated in the X axis labels. QM is an abbreviation for QM9414.

The pH-signalling pathway leading to the activation of PacC in *A. nidulans* consists of the six *pal* genes (*palA*, *palB*, *palC*, *palF*, *palH* and *palI*) together with the components of the ESCRT-complex (for example Vps20, Vps23, Vps24 and Vps32). The *T. reesei* homologues of the *A. nidulans pal*-genes and *vps*-genes were searched from the genome and the behaviour of these genes in the transcriptome data on different pH conditions was studied. Comparison of the three pH conditions showed a significant increase of *pac1* expression with increasing pH (using a fold change log2 > 0.4 and a p-value < 0.01 as a threshold) and a decrease in the expression of *palF* (gene ID 56605) with increasing pH (p-value < 0.05 as a threshold) (Figure 3A). The analysis did not detect significant changes in expression of the other *pal* or *vps* homologues, suggesting a rather steady expression of these genes regardless of pH in *T. reesei*.

In the Δ*pac1* strain, the expression of the *palC* homologue (gene ID 80523) was significantly lower (p-value < 0.01) as compared to the expression of the gene in the parental strain QM9414 (Figure 3B). The result suggests that the *palC* homologue could be, at least in part, under positive PACI-mediated regulation. Furthermore, both *pac1* and *palC* have multiple consensus motifs (GCCARG) in their promoter for putative PACI binding. The expression of *palF* showed the opposite trend by being expressed at a slightly higher level in the Δ*pac1* strain as compared to QM9414 (Figure 3B). However, the difference in the *palF* expression between the strains was not statistically significant.

### The effect of pH on genes encoding carbohydrate active enzymes and on the regulators of cellulase- and hemicellulase-encoding genes

**Figure 4:**
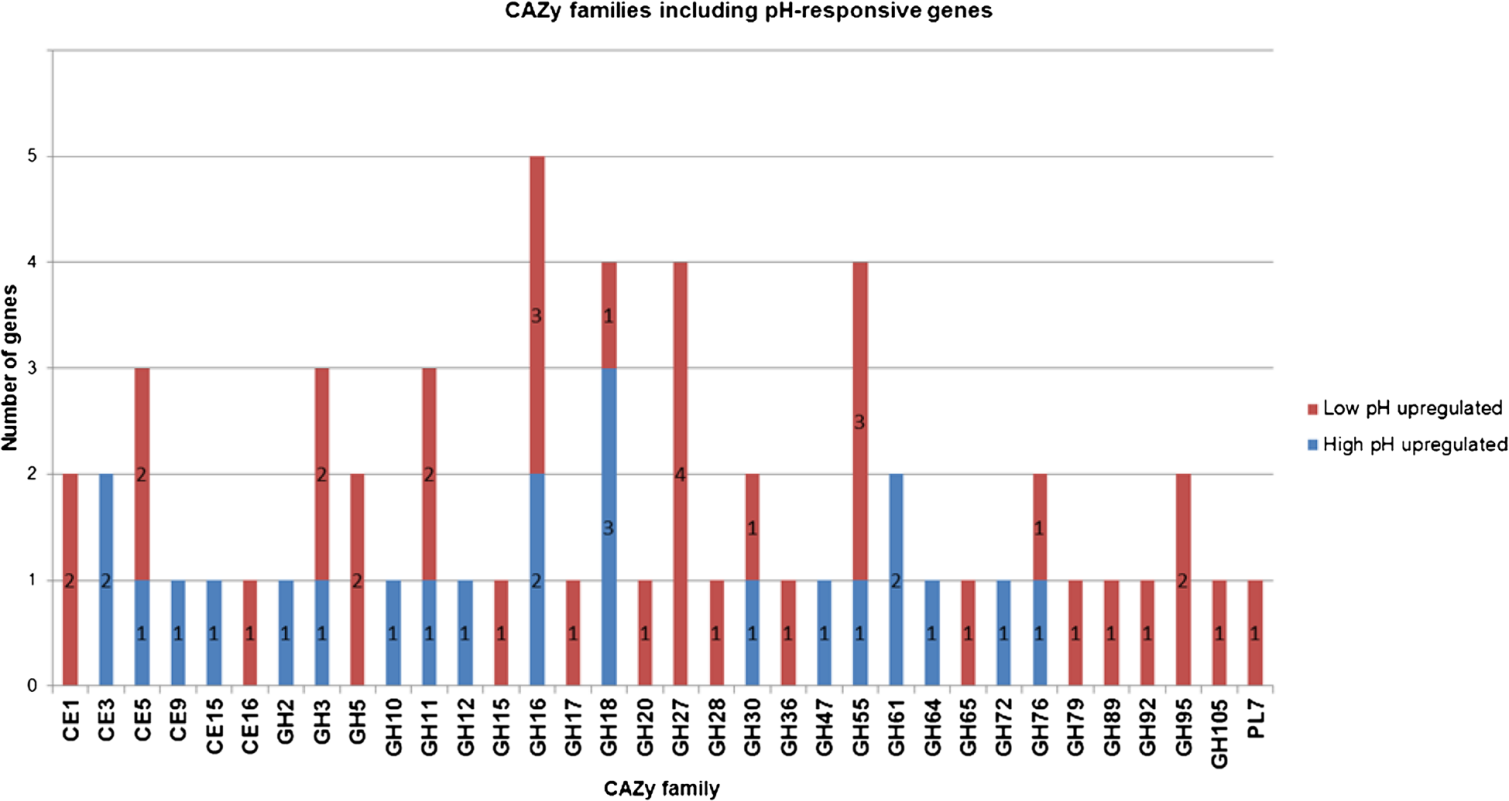
pH-responsive genes encoding glycoside hydrolases, carbohydrate esterases and polysaccharide lyases. The number of pH-responsive genes encoding enzymes of different CAZy families is shown (Genes with a significant difference in expression, p value < 0.01 and fold change log2 > 0.4, detected at both time points of the triplicate cultures, are included).

Due to the fact that *T. reesei* is an exceptionally efficient producer of cellulases and hemicellulases, the effect of pH on the expression of genes encoding carbohydrate active enzymes (CAZy, [31,32]) was studied in more detail (microarray data on the genes encoding carbohydrate active enzymes is presented in Additional file 3). The majority of the enzymes involved in the degradation of plant cell wall material belong to the classes of glycoside hydrolases (GH), carbohydrate esterases (CE) and polysaccharide lyases (PL). Of the pH-responsive genes, 50 are classified as genes encoding glycoside hydrolases, 10 encoding carbohydrate esterases and one encoding a polysaccharide lyase (Figure 4). Of these genes, 23 and 38 were determined to be up-regulated at high pH and at low pH, respectively.

Genes encoding members of the CAZy families GH16, GH18, GH27, and GH55 were especially abundant among the pH-responsive genes, but also a group of genes encoding CAZy enzymes with activities against cellulose or hemicellulose polymers were found among the pH-responsive genes. Especially, many of the genes for xylanolytic enzymes were differentially expressed at different pH. In addition, differential expression within a CAZy family or within a group of genes encoding enzymes with a similar type of activity was detected. Especially, the multiple xylanases and acetylxylan esterases, as well as CAZy families GH3, GH16, GH18 and GH55, include both members that are up-regulated at low or high pH.

Expression of the genes encoding the major cellulases in *T. reesei* (*cbh1, cbh2, egl1* and *egl2*), was not significantly affected under the different pH conditions studied, but endoglucanases of families GH5 and GH12 were among the differentially expressed ones. For details of the CAZy family members encoded by the differentially expressed genes, see Table 1.

**Table 1:** **pH-responsive genes encoding CAZy family enzymes (glycoside hydrolases, carbohydrate esterases, polysaccharide lyases, and auxiliary activities)**

**CAZy family**	**Up-regulated genes at high pH**	**Up-regulated genes at low pH**
AA9	22129 Polysaccharide monooxygenase,	
	31447 Polysaccharide monooxygenase	
CE1		107850 Candidate esterase,
		107268 Candidate S-formylglutathione hydrolase
CE15	123940 *cip2* Glucuronoyl esterase	
CE16		103825 Candidate acetyl esterase
CE3	44366 Candidate esterase,	
	70021 Candidate acetyl xylan esterase	
CE5	54219 Candidate acetyl xylan esterase	44214 *axe2* Candidate acetyl xylan esterase,
		60489 Candidate cutinase
CE9	79671 N-acetyl-glucosamine-6-phosphate deacetylase	
GH3	47268 *bgl3i* Candidate beta-glucosidase	104797 *bgl3j* Candidate beta-glucosidase,
		58450 *xyl3b* Candidate beta-xylosidase
GH5		71554 Beta-1,3-mannanase/endo-beta-1,4-mannosidase,
		82616 *cel5b* Candidate membrane bound endoglucanase
GH10	120229 *xyn3* Endo-beta-1,4-xylanase	
GH11	123818 *xyn2* Endo-beta-1,4-xylanase	74223 *xyn1* Endo-beta-1,4-xylanase,
		112392 *xyn5* Candidate endo-beta-1,4-xylanase
GH30	69276 Candidate endo-beta-1,4-xylanase	3094 Candidate glucan endo 1,6-beta-glucanase
GH12	123232 *egl3/cel12a* Endo-beta-1,4-glucanase	
GH15		65333 Alpha-glycosidase (Glucoamylase and related)
GH16	65406 Candidate cell wall glucanosyltransferase,	49274 Candidate glucan endo-1,3(4)-beta-D-glucosidase,
	55886 Candidate glucan endo-1,3(4)-beta-D-glucosidase	21294 Candidate glucan endo-1,3(4)-beta-D-glucosidase,
		39755 Candidate glucan endo-1,3(4)-beta-D-glucosidase
GH17		39942 Candidate glucan endo-1,3-beta-glucosidase
GH18	43873 *chi18-12* Candidate chitinase,	110317 *chi18-17* Candidate chitinase
	59791 *chi18-15* Candidate chitinase,	
	65162 *endoT* Endo-N-acetyl-beta-D-glucosaminidase	
GH2	102909 Candidate GH2	
GH20		105931 Candidate N-acetyl-beta-hexosaminidase
GH27		72632 *agl1* alpha-galactosidase,
		72704 *agl3* alpha-galactosidase,
		27219 Candidate alpha-galactosidase,
		27259 Candidate alpha-galactosidase
GH28		70186 Polygalacturonase/xylogalacturonan hydrolase
GH36	64827 Candidate raffinose synthase domain protein	
GH47	79960 Candidate alpha-1,2-mannosidase	
GH55	70845 Candidate beta-1,3-glucanase	56418 Candidate beta-1 3-glucanase,
		54242 Candidate beta-1,3-glucanase,
		73248 Candidate exo-1,3-beta-glucanase
GH64	124175 Candidate endo-1,3-beta-glucanase	
GH65		123456 Candidate alpha,alpha-trehalase
GH72	123538 Candidate beta-1 3-glucanosyltransferase	82633 Candidate beta-1 3-glucanosyltransferase
GH76	122495 Candidate alpha-1,6-mannanase	
GH79		71394 Candidate beta-glucuronidase
GH89		58117 Candidate alpha-N-acetylglucosaminidase
GH92		60635 Candidate alpha-1,2-mannosidase
GH95		72488 Candidate alpha-L-fucosidase,
		111138 Candidate alpha-L-fucosidase
GH105		4221 Candidate rhamnogalacturonyl hydrolase
PL7		103033 Candidate alginate lyase
GH		105288

Genes showing differential expression in comparison of different pH conditions (significant difference in expression, with p value < 0.01 and fold change log2 > 0.4, detected at both time points of the triplicate cultures). Gene ID number (JGI, http://genome.jgi-psf.org/Trire2/Trire2.home.html), common name of the gene and the CAZy family and activity of the encoded enzyme are shown.

Expression of relatively few of the genes encoding CAZy enzymes was affected in the ∆*pac1* strain (Table 2). Seven glycoside hydrolase genes and one carbohydrate esterase gene were down-regulated in the ∆*pac1* strain, and one glycoside hydrolase gene was up-regulated. Only two of the glycoside hydrolase genes and one carbohydrate esterase gene had an expression pattern that suggested PACI-mediated regulation. These include genes encoding a candidate β-glucosidase (*bgl3i*), a candidate GH76 α-1,6-mannanase (gene ID 122495) and a candidate CE9 N-acetyl-glucosamine-6-phosphate deacetylase (gene ID 79671). No GH or CE genes under negative PACI regulation were detected with the statistical selection criteria used. However, clustering of the expression data shows an expression pattern expected for PACI-regulated gene for a set of CAZy genes. The vast majority of the PACI-repressed pH-responsive genes were assigned to three different Mfuzz clusters, and PAC1-induced genes to one cluster (Additional file 2). In total 23 CAZy genes were found in these clusters (Table 3).

**Table 2:** **Differential expression of genes encoding CAZy family enzymes (glycoside hydrolases, carbohydrate esterases, polysaccharide lyases, and auxiliary activities) in the ∆** ***pac1*** **strain**

**Gene ID**	**Name**	**CAZy family**	**CAZy annotation**	**Expression at pH6 vs. pH3**	***Expression in the strain ∆pac1 vs. QM9414 at pH6***
47268	bgl3i	GH3	Candidate beta-glucosidase	**pH6 > pH3**	***QM9414 > ∆pacC***
122495		GH76	Candidate alpha-1,6-mannanase	**pH6 > pH3**	***QM9414 > ∆pacC***
79671		CE9	N-acetyl-glucosamine-6-phosphate deacetylase	**pH6 > pH3**	***QM9414 > ∆pacC***
76227	cel3e	GH3	Candidate beta-glucosidase		***QM9414 > ∆pacC***
82235		GH31	Candidate alpha-glucosidase		***QM9414 > ∆pacC***
123226		GH37	Candidate alpha,alpha-trehalase		***QM9414 > ∆pacC***
71532		GH71	Candidate alpha-1 3-glucanase		***QM9414 > ∆pacC***
53542		GH76	Candidate alpha-1,6-mannanase		***QM9414 > ∆pacC***
69123		GH76	Candidate alpha-1,6-mannanase		***∆pacC > QM9414***

Genes showing differential expression in comparison of the strain ∆pac1 and QM9414 (significant difference in expression, with p value < 0.01 and fold change log2 > 0.4, detected at both time points of the triplicate cultures). For comparison the differential expression of the genes at different pH in the parental strain QM9414 is shown. Gene ID number (JGI, http://genome.jgi-psf.org/Trire2/Trire2.home.html), common name and the CAZy family and activity of enzyme encoded by the gene are included in the Table.

**Table 3:** **Clustering of genes encoding CAZy family enzymes (glycoside hydrolases, carbohydrate esterases, polysaccharide lyases, and auxiliary activities) based on the expression array data**

**Gene ID**	**Name**	**CAZy family**	**CAZy annotation**	**Co-clustering with candidate PACI induced genes**	**Co-clustering with cand. PACI repressed genes**
72072		CE1	Candidate esterase	Cluster 49	
44366		CE3	Candidate esterase	Cluster 49	
79671		CE9	N-acetyl-glucosamine-6-phosphate deacetylase	Cluster 49	
108348		GH	Unknown	Cluster 49	
123818	xyn2	GH11	Endo-beta-1,4-xylanase	Cluster 49	
65406		GH16	Candidate cell wall glucanosyltransferase	Cluster 49	
109278		GH24	Candidate lysozyme	Cluster 49	
47268	bgl3i	GH3	Candidate beta-glucosidase	Cluster 49	
77942		GH72	Candidate beta-1 3-glucanosyltransferase	Cluster 49	
122495		GH76	Candidate alpha-1,6-mannanase	Cluster 49	
79921		GH92	Candidate alpha-1,2-mannosidase	Cluster 49	
103825		CE16	Candidate acetyl esterase		Cluster 25
41248		CE3	Candidate acetyl xylan esterase		Cluster 25
65215		CE4	Candidate imidase		Cluster 25
4221		GH105	Candidate rhamnogalacturonyl hydrolase		Cluster 25
73101		GH16	Candidate glucan endo-1,3-1,4-beta-D-glucosidase		Cluster 25
70542		GH16	Candidate b-glycosidase (endo-beta-1,3(4)-beta-D-glucanase)		Cluster 20
110317	chi18-17	GH18	Candidate chitinase		Cluster 25
76852		GH2	Candidate beta-galactosidase/beta-glucuronidase		Cluster 20
72632	agl1	GH27	alpha-galactosidase		Cluster 15
27219		GH27	Candidate alpha-galactosidase		Cluster 15
27259		GH27	Candidate alpha-galactosidase		Cluster 15
70186		GH28	Candidate polygalacturonase/xylogalacturonan hydrolase		Cluster 15
58450	xyl3b	GH3	Candidate beta-xylosidase		Cluster 15
69736		GH30	Candidate glucan endo 1,6-beta-glucanase		Cluster 25
80240	bga1	GH35	Ceta-galactosidase		Cluster 20
73102		GH39	Candidate beta-xylosidase		Cluster 15
73248		GH55	Candidate exo-1,3-beta-glucanase		Cluster 15
69123		GH76	Candidate alpha-1,6-mannanase		Cluster 25
71394		GH79	Candidate beta-glucuronidase		Cluster 25
74198		GH92	Candidate alpha-1,2-mannosidase		Cluster 25
72488		GH95	Candidate alpha-L-fucosidase		Cluster 15
105288		GHNA	Unknown		Cluster 15
69189		PL20	Candidate endo-beta-1,4-glucuronan lyase		Cluster 25

Mfuzz was used for clustering of the microarray expression data, and genes that encode CAZy enzymes and co-cluster with the candidate PACI-regulated genes are listed.

**Figure 5:**
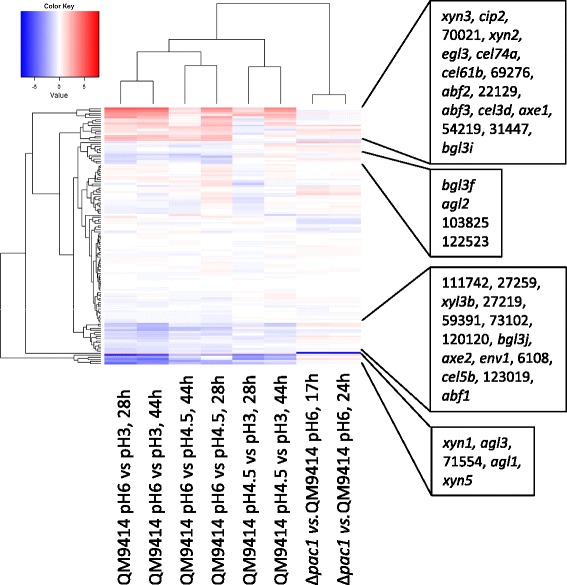
Heat map visualisation of expression data on the genes encoding cellulases and hemicellulases and of the characterized and putative regulatory genes. The colour key indicates the log2 scale fold change of the transcript signals in the comparison between two different pH conditions or between Δpac1 strain and the parental strain QM9414 at a corresponding time point of cultivation. The pH and the cultivation are indicated in the sample names below the heat map. The genes are shown as rows and the samples as columns.

The expression patterns of the genes encoding for the candidate or characterized (*xyr1*, *ace1*, *ace2* and *cre1*) regulators of cellulase and hemicellulase genes were also studied and compared to those of the cellulase- and hemicellulase-encoding genes. The characterized regulators were not pH-regulated according to the Limma test but some of the novel candidate regulatory genes identified during a previous study [33] (genes 111742 (pMH14), 120120 (pMH22) and 123019 (pMH30)) were up-regulated at low pH (Additional file 1). A heatmap representation from the fold changes of the expression levels of the genes encoding cellulolytic and hemicellulolytic enzymes and of the characterized or candidate regulatory genes for these enzyme genes is shown in Figure 5. Genes encoding the components of the pH-signalling pathway were also included. Genes forming the heat map are listed in Additional file 4. The candidate regulatory genes 111742 (pMH14), 120120 (pMH22), 123019 (pMH30) and 122523 (pMH29) [33] group together with the genes up-egulated at low pH. Interestingly, the *ace3* gene encoding a novel regulator of especially cellulase genes [33] cluster together with *cel3c*, a candidate β-xylosidase/α-L-arabinofuranosidase gene (3739), *egl2*, *cbh2*, *swo1*, *egl4* and two candidate regulatory genes (121121 and 56077), indicating similar expression of these genes under the prevailing conditions.

### Co-regulated genomic regions

Mfuzz clustering of the expression profiles was performed in order to study the expression patterns of the genes in more detail and to identify co-regulated groups of genes. Co-regulated genes were also searched for in a genomic scale, identifying genes located close to each other in the scaffolds and being co-regulated. Co-expressed genomic regions were found by searching the genome for neighbouring genes that were assigned to the same Mfuzz cluster (at least 3 genes from 4 consecutive genes belonging to the same Mfuzz cluster) and of which at least one was pH-responsive according to the Limma analysis (in the strain QM9414 and/or in the deletion strain). In total 40 such clusters were found. The pH-responsive members of the clusters are marked in the Additional files 1 and 2.

Genomic clusters of co-regulated pH-responsive genes most probably involved in siderophore biosynthesis were identified. There is a tightly co-regulated genomic region in scaffold 46 containing six genes up-regulated at high pH. The genes are not under PACI regulation according to the criteria used. According to manually curated annotations, these genes putatively encode a non-ribosomal peptide synthase, a siderophore biosynthesis lipase/esterase, an ABC transporter, a siderophore biosynthesis acetylase, an enoyl-CoA hydratase/isomerase and a siderophore iron transporter. A very similar co-regulated cluster of six genes that are up-regulated at high pH and which is located in scaffold 20 includes genes annotated to encode an ABC transporter, an oxidoreductase, an MFS transporter, a siderophore biosynthesis acetylase, a long chain fatty acid acyl-CoA ligase and a non-ribosomal peptide synthase. Genes 67026 and 67189 belonging to this cluster are not pH-responsive according to the Limma analysis, although the *T. virens* homologues for these genes have been shown to be up-regulated at alkaline pH [34].

An additional gene cluster most probably involved in siderophore biosynthesis but not passing the criteria set for the co-regulated genomic regions is located in scaffold 31. The cluster includes a gene homologous to the *T. virens* siderophore biosynthesis gene *sidA* which is a gene expressed at high pH, as is the *T. virens* counterpart. Similarly, the homologue of *T. virens* non-ribosomal peptide synthase gene (*tex10*) is clustered with the siderophore biosynthesis gene but is not co-regulated with it (and therefore is not assigned to the same Mfuzz-cluster).

Three clusters possibly involved in cellulase signalling were found. In scaffold 17, a cluster of three genes that are up-regulated at high pH, included the *ooc1* gene encoding a secreted protein suggested to be involved in cellulase signal transduction [35] together with a gene homologous to *ooc1* and a gene of unknown function. *ooc1* encodes a small acidic protein the expression of which is abundant on cellulose in strain QM9414 [35]. A cluster of three genes from scaffold 13 includes a major facilitator superfamily transporter gene 79202 possibly involved in cellulase signal transduction [36]. The two other genes belonging to the cluster encode proteins of unknown function. Only one of the unknown genes is pH-responsive (up-regulated at high pH), whereas expression of the other genes is not significantly affected by pH. These three genes belong to the same Mfuzz cluster as the majority of the genes induced by PACI.

Another cluster of genes up-regulated at low pH in scaffold 29 includes a gene encoding GRDI that is a putative dehydrogenase associated with cellulase signal transduction [37]. The other members of the cluster include four genes with unknown functions and a fungal transcriptional regulatory protein (pMH14 [33]).

## Discussion

The transcriptome of *T. reesei* at different extracellular pH values was studied in order to elucidate the role of pH regulation in a saprotrophic cellulolytic fungus. Ambient pH was found to be an important determinant of *T. reesei* gene expression. The gene groups abundant among the pH-responsive genes included e.g. genes encoding transporters, exported enzymes, signalling-related functions, regulatory proteins, and proteins involved in various metabolic reactions. Especially, the abundance of genes encoding the extracellular glycoside hydrolases among the pH-responsive genes indicates that adaptation to changing ambient pH conditions is an important determinant of the success of a saprotrophic fungus.

The regulatory mechanisms induced by ambient pH were also subjected to the study by deletion of the regulatory gene *pac1*, and analysis of the expression level of the genes encoding the components in the pH signalling pathway. In *T. reesei*, the expression patterns of the *pac1* deletion strain and comparison of the different pH conditions suggested a possible role for PACI in their regulation pH-dependent regulation of *pal* genes have been studied in the fungus *Magnaporthe oryzae* [38]. The only *pal* gene influenced by pH and by the deletion of the *pacC* paralogue was MOPALF (a homologue of *palF*). The expression of the MOPALF gene decreased significantly with increasing pH from 5 to 8. Deletion of MOPACC (a homologue of *pac1*) indicated MOPACC-mediated repression of MOPALF. In previous studies, it was concluded that expression of the genes of the Pal-signalling pathway is not regulated by ambient pH in the fungi *A. nidulans* and *A. niger* [39,40]. These contradictory results suggest that either these fungi use partially different mechanisms for the regulation of pH signalling or is difficult to detect using traditional significance tests.

The expression patterns of the genes encoding enzymes involved in the degradation of cellulose and hemicellulose polymers were studied in more detail. Especially, the genes encoding hemicellulases appeared to be differentially regulated depending on the ambient pH. As expected for genes that encode for enzymes with related activities but functioning at different pH, the gene set contained both genes that were up-regulated at low pH and genes that were up-regulated at high pH. The xylanase genes of T. reesei are a good example of this. The genome of *T. reesei* encodes four characterized and two candidate xylanases involved in the degradation of the xylan chain of hemicellulose. The *xyn2*, *xyn3* and a candidate GH30 xylanase gene (69276) are abundantly expressed at high pH, whereas *xyn1* and *xyn5* are more highly expressed at a lower pH. In addition, PACI was suggested to have a slight inducing effect on *xyn2* according to the Mfuzz clustering, and a putative PACI binding site was found on the promoter of *xyn2*. The pH optima for the characterized *T. reesei* xylanases have been determined and the data support the results of the transcriptional analysis. XYNIII has a rather high pH optimum (6–6.5), whereas the pH optima of XYNI and XYNII are 4.0-4.5 and 4.0-6.0, respectively [41-44]. In lactose medium, XYNI and XYNIII are preferentially produced at pH4 and pH6, respectively [45], and XYNII is produced under both pH conditions.

Although a variety of CAZy genes respond to change of pH, only a few are clearly under PACI regulation. It is also possible that other regulatory mechanisms have a stronger effect and mask the effect of PACI-regulation. For example, the promoter of the *Aspergillus tubingensis* xylanase gene *xlnA* contains overlapping binding sites for the activator XlnR and PacC, suggesting that PacC could be competing with the activator [46]. Several other activators and repressors besides XYRI [47] are involved in the regulation of cellulase and hemicellulase genes of *T. reesei*. These include for example ACEI, ACEII and CREI [48-51]. Of the novel candidate regulatory genes identified during a previous study [33], the genes 111742 (pMH14), 120120 (pMH22) and 123019 (pMH30) were up-regulated at low pH. Over-expression of these genes has been shown to result in slightly declined production of cellulase activity [33]. Interestingly, the novel regulator of especially cellulase genes, *ace3* [33], was assigned to the same Mfuzz cluster and/or heat map branch as several genes that were already previously shown to cluster together with *ace3* in a dataset from a transcriptional analysis with several different inducing carbon sources [33]. These genes included *egl2*, *egl3*, *cbh2*, *swo1*, *cip2*, *xyn3*, *cel1b* and *cel3c*. These results indicate that regardless of the environmental conditions (different inducing substrates or different ambient pH), the genes most probably under *ace3* regulation are co-expressed with *ace3*.

The genomic cluster containing the *grd1* gene which is believed to be involved in cellulase signal transduction also included the gene 111742 encoding a transcriptional regulator. Both genes were more highly expressed at low pH as compared to high pH. The over-expression of gene 111742 (pMH14) had a negative effect on the production of cellulase activity and especially on endoglucanase activity [33]. GRDI, however, has been shown to have a positive effect on cellulase activity [37]. These results make this genomic cluster an interesting target for the study of cellulase signalling in *T. reesei*.

## Conclusions

In this study, the pH-responsive genes from the genome of *T. reesei* were identified, including genes affected by the regulator of ambient pH sensing, PACI. The effect of ambient pH on the genes of the pH-signalling cascade leading to activation of PACI was investigated and novel information was gained from the pH-regulation of the carbohydrate active enzyme genes and especially genes encoding cellulose- and hemicellulose-degrading enzymes. In addition, several co-regulated genomic clusters responding to changes in ambient pH were identified.

## Materials and methods

### Strains, media and culture conditions

Escherichia coli DH5α (*fhuA2* Δ*(argF-lacZ)U169 phoA glnV44 Φ80* Δ*(lacZ)M15 gyrA96 recA1 relA1 endA1 thi-1 hsdR17*) was used for propagation of the plasmids and *Saccharomyces cerevisiae* FY834 (*MAT*α *his3*Δ*200 ura3-52 leu2*Δ*1 lys2*Δ*202 trp1*Δ*63*) for yeast recombinational cloning. *Trichoderma reesei* QM9414 (ATCC 26921, VTT-D-74075) was obtained from VTT Culture Collection and maintained on potato-dextrose agar plates (Difco). Spore suspensions were prepared by cultivating the fungus on potato-dextrose plates for 5 days, after which the spores were dislodged, suspended in a buffer containing 0.8% NaCl, 0.025% Tween20 and 20% glycerol, filtered through cotton and stored at −80°C. In order to collect mycelia for DNA isolation, the fungus was grown in a medium containing 0.2% proteose peptone (BD Biosciences), 2% glucose, 7.6 g/l (NH_4_)_2_SO_4_, 15.0 g/l KH_2_PO_4_, 2.4mM MgSO_4_.7H_2_O, 4.1 mM CaCl_2_.H_2_O, 3.7 mg/l CoCl_2_, 5 mg/l FeSO_4_.7H_2_O, 1.4 mg/l ZnSO_4_.7H_2_O, 1.6 mg/l MnSO_4_.7H_2_O, pH 4.8. Flask inoculum for the bioreactor cultivations was prepared by cultivating 8 × 10^7^ spores for 3 days in 400 ml of medium containing 25 g/l Avicel cellulose (Fluka BioChemika), 6 g/l proteose peptone (BD Biosciences), 7.6 g/l (NH_4_)_2_SO_4_, 15.0 g/l KH_2_PO_4_, 2.4 mM MgSO_4_.7H_2_O, 4.1 mM CaCl_2_.H_2_O, 3.7 mg/l CoCl_2_, 5 mg/l FeSO_4_.7H_2_O, 1.4 mg/l ZnSO_4_.7H_2_O, 1.6 mg/l MnSO_4_.7H_2_O, pH adjusted to 5.2 with KOH. Bioreactor medium contained 25 g/l Avicel cellulose, 6 g/l proteose peptone, 13.6 g/l (NH_4_)_2_SO_4_, 4 g/l KH_2_PO_4_, 0.2 ml/l Tween80, 0.6 g/l MgSO_4_.7H_2_O, 0.8 g/l CaCl_2_.H_2_O, 3.7 mg/l CoCl_2_, 5 mg/l FeSO_4_.7H_2_O 1.4 mg/l ZnSO_4_.7H_2_O, 1.6 mg/l MnSO_4_.7H_2_O and 1 ml/l of antifoam agent (Dow corning 1500). pH of the medium was adjusted before inoculation to pH3, 4.5 or 6. The bioreactor containing 0.9 l of medium was inoculated with 0.1 l of flask inoculum. Bioreactor cultivations were performed in Sartorius Q plus reactors at 28°C with a dissolved oxygen saturation level of >30%, agitation of 500–1200 rpm and a constant air flow of 0.6 l/min. pH was controlled with 15% KOH and 15% H_3_PO_4_. Samples from the cultivations of the QM9414 strain at different pH values were collected at time points of approximately 8 h, 18 h, 28 h, 44 h, 68 h, 93 h and 119 h. Samples from the cultivation of the QM9414 and ∆pac1 strains at pH6 were collected at time points 17 h, 24 h, 40.5 h, 69 h, 95.3 h, 116.5 h and 139.3 h.

### Construction of the *pac1* deletion strain

Deletion cassette for the knock-out of the *pac1* gene was constructed by yeast recombination cloning [52] in the pRS426 plasmid [53]. The construct contained a hygromycin resistance cassette flanked by 1890 bp fragments from 5′ and 3′ ends of the *pac1* gene. Fragment 5′ from the *pac1* open reading frame was PCR amplified with the oligos 5′- GTAACGCCAGGGTTTTCCCAGTCACGACGGTTTAAACCCACCTCACCAGCCTTTGGTTTGCA-3′ and 5′- ACCGGGATCCACTTAACGTTACTGAAATCGGAAGGTCTTGGCGGTCTTGGCATT-3′. Fragment 3′ from the *pac1* open reading frame was PCR amplified with the oligos 5′- ATGCCAGAAAGAGTCACCGGTCACTGTACTGAATGAACATTCTTCAACATAACA-3′and 5′-GCGGATAACAATTTCACACAGGAAACAGCGTTTAAACCGCAGCAGCAGCATTGCTTGGGCGG-3′. Hygromycin resistance cassette was PCR amplified with the oligos 5′- AGACAATGCCAAGACCGCCAAGACCTTCCGATTTCAGTAACGTTAAGTG-3′and 5′ TGGGTGTTATGTTGAAGAATGTTCATTCAGTACAGTGACCGGTGACTCT-3′. All the PCR products were purified and combined with the vector backbone. The resulting plasmid was digested with *PmeI* and *SspI* enzymes (New England biolabs) and the deletion cassette was transformed to *T. reesei* QM9414 by polyethylene glycol mediated protoplast transformation [54]. The hygromycin resistant transformants were initially selected from plates containing 150 μg ml^−1^ of hygromycin B (Calbiochem). Stable transformants were obtained by streaking on plates containing 125 μg ml^−1^ of hygromycin B for two successive rounds. Single colonies resulting from uninuclear transformants were obtained by plating dilutions of spore suspensions. Transformants with the expression cassette at the correct locus were identified by PCR and integration of only one copy of the cassette was further verified by Southern blot analysis. Genomic DNA for Southern blot was isolated using Easy-DNA Kit (Invitrogen) according to the manufacturer’s instructions. Southern blotting and hybridisation on nitrocellulose filters (Hybond N, GE Healthcare) were carried out according to standard procedures [55]. The signals were detected using a phosphorimager (Typhoon imager, GE Healthcare). The deletion of the *pac1* gene was confirmed by Norhern blot analysis. Total RNA was isolated from the mycelium samples using the Trizol™ Reagent (Invitrogen Life Technologies, Carlsbad, CA, USA) essentially according to the manufacturer’s instructions. Northern blotting and hybridisation on nitrocellulose filters (Hybond N, GE Healthcare) were carried out according to standard procedures [55].

### Sample preparation and microarray analytics

Mycelial samples collected at the time points of 28 and 44 hours from the cultures of QM9414 at pH3, 4.5 and 6, as well as samples collected at the time points of 17 and 24 hours from the parallel cultures of the strain Δpac1 and QM9414, were subjected to transcriptome analysis. Frozen mycelium was ground under liquid nitrogen, and total RNA was isolated with Trizol reagent according to the manufacturer’s instructions. RNA was subsequently purified using RNeasy Mini Kit (Qiagen, Hilden, Germany) and RNA concentration was measured using NanoDrop ND-1000 (NanoDrop Technologies Inc. Wilmington, DE, USA). Integrity of the isolated RNA was verified using an Agilent 2100 Bioanalyzer (Agilent Technologies, Palo Alto, CA, USA).

Processing of the RNA samples for the microarray analysis was performed essentially according to the instructions from RocheNimblegen. After synthesising the double-stranded cDNA using Superscript Double-Stranded cDNA synthesis Kit (Invitrogen), the integrity of the double-stranded cDNA was analysed using an Agilent 2100 Bioanalyzer. The double-stranded cDNA was labelled with Cy3 fluorescent dye, after which it was hybridized to microarray slides (Roche‐NimbleGen, Inc., Madison, WI, USA) and the slides were scanned using a Roche NimbleGen Microarray scanner according to the instructions of the manufacturer. The probe design and manufacturing of the microarray slides was carried out by RocheNimbleGen using an array design based on the *T. reesei* genome version 2.0 [24]. The design includes six 60mer probes for each of the genes.

The data was pre-processed using the R package Oligo and differentially expressed genes were identified with the package Limma [27,28,56]. Differentially expressed genes were identified by comparing the signals from the different pH-conditions and between the different strains at the corresponding time point. pH6 samples were compared to pH3 and pH4.5 samples and pH4.5 samples were compared to pH3 samples. Signals from the cultivation of the ∆pac1 strain were compared to those of the QM9414 strain from the same cultivation conditions. Three biological replicates were analysed for each condition and each time point. The threshold used for statistical significance was p-value <0.01 and log2-scale fold change >0.4.

## Additional files

Additional file 1:
**Transcriptional profiling data of the** **pH-responsive** **genes.** Gene identifiers are as in the *T. reesei* database version 2.0 and 1.2 (in the latter, v1_2 precedes the gene ID) or as in [26]. Signal intensities (log2 scale), fold changes (log2 scale) and significance test (R package limma, p < 0.01, log2 fold change > 0.4) are shown for the genes from two different time points of cultivations. Colour scales of yellow, red and green indicate different intensities of signals, red representing the strongest and green the weakest signals. The intensity of the red and blue colour indicates the strength of positive and negative fold change, respectively. In the significance test, 1 indicates induction and −1 repression. QM9414II indicates the control culture (QM9414) for ∆pac1. Annotations are shown according to the Eukaryotic orthologous group classification and as the manual annotations from the JGI *T. reesei* 2.0 database. Functional Interpro domain identifiers are as in the InterPro database. Annotations of glycoside hydrolase, carbohydrate esterase and polysaccharide lyase genes are as in [30]. Additional file 2:
**Transcriptional profiling data of the genes affected by the** ***pac1*** **deletion.** Gene identifiers are as in the *T. reesei* database version 2.0 and 1.2 (in the latter, v1_2 precedes the gene ID) or as in [26]. Signal intensities (log2 scale), fold changes (log2 scale) and significance test (R package limma, p < 0.01, log2 fold change > 0.4) are shown for the genes from two different time points of cultivations. Colour scales of yellow, red and green indicate different intensities of signals, red representing the strongest and green the weakest signals. The intensity of the red and blue colour indicates the strength of positive and negative fold change, respectively. In the significance test, 1 indicates induction and −1 repression. QM9414II indicates the control culture (QM9414) for ∆pac1. Annotations are shown according to the Eukaryotic orthologous group classification and as the manual annotations from the JGI T. reesei 2.0 database. Functional Interpro domain identifiers are as in the InterPro database. Annotations of glycoside hydrolase, carbohydrate esterase and polysaccharide lyase genes are as in [30]. Additional file 3:
**Transcriptional profiling data of the CAZy genes.** Gene identifiers are as in the *T. reesei* database version 2.0 and 1.2 (in the latter, v1_2 precedes the gene ID) or as in [26]. Signal intensities (log2 scale), fold changes (log2 scale) and significance test (R package limma, p < 0.01, log2 fold change > 0.4) are shown for the genes from two different time points of cultivations. Colour scales of yellow, red and green indicate different intensities of signals, red representing the strongest and green the weakest signals. The intensity of the red and blue colour indicates the strength of positive and negative fold change, respectively. In the significance test, 1 indicates induction and −1 repression. QM9414II indicates the control culture (QM9414) for ∆pac1. Annotations of the genes are as in [30]. Additional file 4:
**Genes from the heat map visualisation.** Gene identifiers are as in the *T. reesei* database version 2.0. Annotations are shown according to the Eukaryotic orthologous group classification and as the manual annotations from the JGI *T. reesei* 2.0 database. Functional Interpro domain identifiers are as in the InterPro database. Annotations of glycoside hydrolase, carbohydrate esterase and polysaccharide lyase genes are as in [30]. References for the characterized and candidate regulatory genes of the cellulase and hemicellulase genes are shown.

## Abbreviations

CAZy: Carbohydrate active enzyme
CBHI: Cellobiohydrolase 1
DNS: 2-hydroxy-3,5-dinitrobenzoic acid
EGI: endo-β-1,4-glucanase 1
JGI: Joint Genome Institution
KOG: Eukaryotic orthologous groups
MU: Methyl umbelliferone
MUL: 4-methyl umbelliferyl-β-D-lactoside

## References

[1] Peñalva MA, Tilburn J, Bignell E, Arst HN (2008). Ambient pH gene regulation in fungi: making connections. Trends Microbiol.

[2] Herranz S, Rodríguez JM, Bussink H-J, Sánchez-Ferrero JC, Arst HN, Peñalva MA (2005). Arrestin-related proteins mediate pH signaling in fungi. Proc Natl Acad Sci U S A.

[3] Calcagno-Pizarelli AM, Negrete-Urtasun S, Denison SH, Rudnicka JD, Bussink H-J, Múnera-Huertas T (2007). Establishment of the ambient pH Signaling complex in *Aspergillus nidulans*: PalI assists plasma membrane localization of PalH. Eukaryot Cell.

[4] Hervás-Aguilar A, Galindo A, Peñalva MA (2010). Receptor-independent ambient pH signaling by ubiquitin attachment to fungal arrestin-like PalF. J Biol Chem.

[5] Galindo A, Calcagno-Pizarelli AM, Arst HN, Peñalva MÁ (2012). An ordered pathway for the assembly of fungal ESCRT-containing ambient pH signalling complexes at the plasma membrane. J Cell Sci.

[6] Diez E, Alvaro J, Espeso EA, Rainbow L, Suarez T, Tilburn J (2002). Activation of the *Aspergillus* PacC zinc finger transcription factor requires two proteolytic steps. EMBO J.

[7] Hervás-Aguilar A, Rodríguez JM, Tilburn J, Arst HN, Peñalva MA (2007). Evidence for the direct involvement of the proteasome in the proteolytic processing of the *Aspergillus nidulans* zinc finger transcription factor PacC. J Biol Chem.

[8] Espeso EA, Tilburn J, Sánchez-Pulido L, Brown CV, Valencia A, Arst HN (1997). Specific DNA recognition by the *Aspergillus nidulans* three zinc finger transcription factor PacC. J Mol Biol.

[9] Tilburn J, Sarkar S, Widdick DA, Espeso EA, Orejas M, Mungroo J (1995). The *Aspergillus* PacC zinc finger transcription factor mediates regulation of both acid- and alkaline-expressed genes by ambient pH. EMBO J.

[10] Caddick MX, Brownlee AG, Arst HN (1986). Regulation of gene expression by pH of the growth medium in *Aspergillus nidulans*. Mol Gen Genet.

[11] Arst HN, Bignell E, Tilburn J (1994). Two new genes involved in signalling ambient pH in *Aspergillus nidulans*. Mol Gen Genet.

[12] Vankuyk PA, Diderich JA, MacCabe AP, Hererro O, Ruijter GJG, Visser J (2004). *Aspergillus niger* mstA encodes a high-affinity sugar/H+ symporter which is regulated in response to extracellular pH. Biochem J.

[13] Maccabe AP, Orejas M, Pérez-González JA, Ramón D (1998). Opposite patterns of expression of two *Aspergillus nidulans* xylanase genes with respect to ambient pH. J Bacteriol.

[14] Gielkens M, González-Candelas L, Sánchez-Torres P, van de Vondervoort P, de Graaff L, Visser J (1999). The *abfB* gene encoding the major a-L-arabinofuranosidase of *Aspergillus nidulans*: nucleotide sequence, regulation and construction of a disrupted strain. Microbiology.

[15] Ke R, Haynes K, Stark J (2013). Modelling the activation of alkaline pH response transcription factor PacC in *Aspergillus nidulans*: involvement of a negative feedback loop. J Theor Biol.

[16] Penttilä M, Limón C, Nevalainen H (2004). Molecular Biology of *Trichoderma* and Biotechnological Applications. Mycol Handb fungal Biotechnol.

[17] Saloheimo M, Pakula TM (2012). The cargo and the transport system: secreted proteins and protein secretion in *Trichoderma reesei* (*Hypocrea jecorina*). Microbiology.

[18] Aro N, Pakula T, Penttilä M (2005). Transcriptional regulation of plant cell wall degradation by filamentous fungi. FEMS Microbiol Rev.

[19] Seiboth B, Herold S, Kubicek C, Wang X, Chen J, Quinn P (2012). Metabolic Engineering of Inducer Formation for Cellulase and Hemicellulase Gene Expression in *Trichoderma Reesei*. Reprogramming Microb Metab Pathways.

[20] Martinez D, Berka RM, Henrissat B, Saloheimo M, Arvas M, Baker SE (2008). Genome sequencing and analysis of the biomass-degrading fungus *Trichoderma reesei* (syn. *Hypocrea jecorina*). Nat Biotechnol.

[21] Bailey M, Buchert J, Viikari L (1993). Effect of pH on production of xylanase by *Trichoderma reesei* on xylan- and cellulose-based media. Appl Microbiol Biotechnol.

[22] Li C, Yang Z, He Can Zhang R, Zhang D, Chen S, Ma L (2013). Effect of pH on cellulase production and morphology of *Trichoderma reesei* and the application in cellulosic material hydrolysis. J Biotechnol.

[23] Adav SS, Ravindran A, Chao LT, Tan L, Singh S, Sze SK (2011). Proteomic analysis of pH and strains dependent protein secretion of *Trichoderma reesei*. J Proteome Res.

[24] v2.0 T reesei database: [http://genome.jgi-psf.org/Trire2/Trire2.home.html]

[25] v1.0 T reesei database: [http://genome.jgi-psf.org/trire1/trire1.home.html]

[26] Arvas M, Haiminen N, Smit B, Rautio J, Vitikainen M, Wiebe M (2010). Detecting novel genes with sparse arrays. Gene.

[27] Bioconductor open source software for bioinformatics: http://www.bioconductor.org/.

[28] Smyth GK, Michaud J, Scott HS (2005). Use of within-array replicate spots for assessing differential expression in microarray experiments. Bioinformatics.

[29] Kumar L, Futschik ME (2007). Mfuzz: a software package for soft clustering of microarray data. Bioinformation.

[30] Häkkinen M, Arvas M, Oja M, Aro N, Penttilä M, Saloheimo M (2012). Re-annotation of the CAZy genes of *Trichoderma reesei* and transcription in the presence of lignocellulosic substrates. Microb Cell Fact.

[31] Cantarel BL, Coutinho PM, Rancurel C, Bernard T, Lombard V, Henrissat B (2009). The Carbohydrate-Active EnZymes database (CAZy): an expert resource for Glycogenomics. Nucleic Acids Res.

[32] Database CAZy: [http://www.cazy.org]

[33] Häkkinen M, Valkonen MJ, Westerholm-Parvinen A, Aro N, Arvas M, Vitikainen M (2014). Screening of candidate regulators for cellulase and hemicellulase production in *Trichoderma reesei* and identification of a factor essential for cellulase production. Biotechnol Biofuels.

[34] Trushina N, Levin M, Mukherjee PK, Horwitz BA (2013). PacC and pH-dependent transcriptome of the mycotrophic fungus *Trichoderma virens*. BMC Genomics.

[35] Schmoll M, Kubicek CP (2005). *ooc1*, a unique gene expressed only during growth of *Hypocrea jecorina* (anamorph: *Trichoderma reesei*) on cellulose. Curr Genet.

[36] Porciuncula De Oliveira J, Furukawa T, Shida Y, Mori K, Kuhara S, Morikawa Y (2013). Identification of major facilitator transporters involved in cellulase production during lactose culture of *Trichoderma reesei* PC-3-7. Biosci Biotechnol Biochem.

[37] Schuster A, Kubicek CP, Schmoll M (2011). Dehydrogenase GRD1 represents a novel component of the cellulase regulon in *Trichoderma reesei* (*Hypocrea jecorina*). Appl Environ Microbiol.

[38] Landraud P, Chuzeville S, Billon-Grande G, Poussereau N, Bruel C (2013). Adaptation to pH and role of PacC in the rice blast fungus *Magnaporthe oryzae*. PLoS One.

[39] Andersen MR, Lehmann L, Nielsen J (2009). Systemic analysis of the response of *Aspergillus niger* to ambient pH. Genome Biol.

[40] Negrete-Urtasun S, Reiter W, Diez E, Denison SH, Tilburn J, Espeso EA (1999). Ambient pH signal transduction in *Aspergillus*: completion of gene characterization. Mol Microbiol.

[41] Tenkanen M, Puls J, Poutanen K (1992). Two major xylanases of *Trichoderma reesei*. Enzyme Microb Technol.

[42] Torronen A, Mach RL, Messner R, Gonzalez R, Kalkkinen N, Harkki A (1992). The two major xylanases from *Trichoderma reesei*: characterization of both enzymes and genes. Nat Biotechnol.

[43] Xu J, Takakuwa N, Nogawa M, Okada H, Okada H (1998). A third xylanase from *Trichoderma reesei* PC-3-7. Appl Microbiol Biotechnol.

[44] Wang J, Zeng D, Mai G, Liu G, Yu S (2013). Homologous constitutive expression of Xyn III in *Trichoderma reesei* QM9414 and its characterization. Folia Microbiol (Praha).

[45] Xiong H, von Weymarn N, Leisola M, Turunen O (2004). Influence of pH on the production of xylanases by *Trichoderma reesei* Rut C-30. Process Biochem.

[46] Graaff LK, Broeck HC, Ooijen AJJ, Visser J (1994). Regulation of the xylanase-encoding *xlnA* gene of *Aspergilius tubigensis*. Mol Microbiol.

[47] Stricker AR, Grosstessner-Hain K, Wurleitner E, Mach RL (2006). Xyr1 (xylanase regulator 1) regulates both the hydrolytic enzyme system and D-xylose metabolism in *Hypocrea jecorina*. Eukaryot Cell.

[48] Aro N, Saloheimo A, Ilmén M, Penttilä M (2001). ACEII, a novel transcriptional activator involved in regulation of cellulase and xylanase genes of *Trichoderma reesei*. J Biol Chem.

[49] Aro N, Ilmen M, Saloheimo A, Penttilä M (2003). ACEI of *Trichoderma reesei* is a repressor of cellulase and xylanase expression. Appl Environ Microbiol.

[50] Saloheimo A, Aro N, Ilmén M, Penttilä M (2000). Isolation of the *ace1* gene encoding a Cys2-His2 transcription factor involved in regulation of activity of the cellulase promoter *cbh1* of *Trichoderma reesei*. J Biol Chem.

[51] Ilmén M, Thrane C, Penttilä M (1996). The glucose repressor gene *cre1* of *Trichoderma*: isolation and expression of a full-length and a truncated mutant form. Mol Gen Genet.

[52] Colot HV, Park G, Turner GE, Ringelberg C, Crew CM, Litvinkova L (2006). A high-throughput gene knockout procedure for *Neurospora* reveals functions for multiple transcription factors. Proc Natl Acad Sci.

[53] Christianson TW, Sikorski RS, Dante M, Shero JH, Hieter P (1992). Multifunctional yeast high-copy-number shuttle vectors. Gene.

[54] Penttilä M, Nevalainen H, Rättö M, Salminen E, Knowles J (1987). A versatile transformation system for the cellulolytic filamentous fungus *Trichoderma reesei*. Gene.

[55] Sambrook J, Fritsch E, Maniatis T (1989). Molecular Cloning: A Laboratory Manual.

[56] Bolstad BM, Irizarry RA, Åstrand M, Speed TP (2003). A comparison of normalization methods for high density oligonucleotide array data based on variance and bias. Bioinformatics.

